# The Electronic Health Insurance Card for Asylum-Seekers in Berlin: Effects on the Local Health System

**DOI:** 10.34172/ijhpm.2021.34

**Published:** 2021-04-17

**Authors:** Nora Gottlieb, Vanessa Ohm, Miriam Knörnschild

**Affiliations:** ^1^Department of Health Care Management, Technical University Berlin, Berlin, Germany.; ^2^Department of Population Medicine and Health Services Research, School of Public Health, Bielefeld University, Bielefeld, Germany.; ^3^Berlin School of Public Health, Berlin, Germany.

**Keywords:** Access to Healthcare, Asylum-Seekers, Cost Analysis, Germany, Health Policy, Mixed-Methods

## Abstract

**Background:** In debates on asylum-seekers’ access to healthcare it is frequently claimed that restrictions are necessary to prevent unduly high health service utilization and costs. Within Germany, healthcare provision for asylum-seekers varies across the different states. Berlin’s authorities removed some barriers to healthcare for asylum-seekers by introducing an electronic health insurance card (HIC) in 2016. We used the HIC introduction in Berlin as an opportunity to investigate the effects of improved healthcare access for asylum-seekers on the local health system.

**Methods:** The study applied a mixed-methods design. A cost analysis compared expenses for outpatient and inpatient health services for asylum-seekers before and after the HIC introduction, based on aggregate claims data and information on expenses for humanitarian healthcare provision that were retrieved from the Berlin authorities. Semi-structured interviews with 12 key informants explored organizational effects like administrative workloads and ethical dilemmas for staff. We performed a content analysis and used respondent validation to enhance the accuracy and trustworthiness of our results.

**Results:** The HIC has reduced bureaucratic complexity and administrative workloads; it has enabled unprecedented financial transparency and control; and it has mitigated ethical tensions. All the while, average per person expenses for outpatient health services have declined since the HIC introduction. However, our cost analysis also indicates a rise in the utilization and costs of inpatient care.

**Conclusion:** The HIC introduction in Berlin suggests that the removal of barriers to healthcare for asylum-seekers can create win-win-situations by reducing administrative workloads, advancing financial transparency, and mitigating ethical tensions, whilst cutting the costs of outpatient healthcare provision. Removing barriers to healthcare thus appears to be a more prudent policy choice than maintaining mechanisms of restriction and control. However, high inpatient care utilization and costs warrant further research.

## Background

**Key Messages**

**Implications for policy makers**

- The Berlin Senate eased asylum-seekers’ access to healthcare by introducing an electronic health insurance card (HIC) in 2016. Such policy change often raises concerns of increased healthcare utilization and costs.
- This study examined effects of the HIC introduction on the local health system, namely cost control and containment.
- It shows decreasing expenses for outpatient healthcare. This finding contradicts the argument that restrictions were necessary to prevent asylum-seeking patients from overusing health services.
- Moreover, the HIC has reduced administrative workloads, enabled unprecedented financial transparency, and mitigated ethical tensions for staff. High expenses for inpatient care persist, however, and warrant further investigation.
- If the goal is to prevent extra burdens on the health system and thus safeguard scarce resources, we recommend the removal of barriers to healthcare.

**Implications for the public**

The public has a legitimate interest that its resources are distributed in transparent and cost-effective ways, and in line with professional and ethical standards. Restrictions on asylum-seekers’ healthcare access are often described as a necessary means to prevent undue healthcare utilization and costs. We wanted to find out if restrictions are a viable strategy to prevent costs and burdens on the health system. To this end, we studied the effects of the introduction of an electronic health insurance card (HIC) for asylum-seekers on the health system in Berlin. With the HIC, some barriers to healthcare were removed. Our study found that the HIC has various advantages for the health system: It has reduced administrative workloads, enabled unprecedented financial transparency, and mitigated ethical tensions for staff. At the same time, expenses for outpatient health services decreased. This means that the previous restrictions did not achieve their goal of containing costs and burdens on the health system. On the contrary, the example of the HIC introduction in Berlin shows that removing barriers to healthcare for asylum-seekers can create win-win-situations for the health system, patients, and public finances.

In 2015, following the arrival of large numbers of migrants in irregular situation, the Berlin Senate changed its health policy for asylum-seekers with the declared goals of easing access and reducing bureaucracy. It decreed the introduction of an electronic health insurance card (HIC) for asylum-seekers, which replaced the previous healthcare vouchers (HVs) in 2016.^1^ At the time, the governments of several German states considered a policy change from HVs to HICs. The surrounding discussions picked up a long-standing debate about health policies for asylum-seekers. In this debate some policy-makers justify restrictions on asylum-seekers’ access to healthcare with the need to prevent overuse and thus safeguard public resources; while others argue for equitable healthcare access.^2^ This study assesses the effects of the HIC introduction in Berlin on the local health system toward the ultimate goal of expanding the evidential basis for policy-making.

###  Healthcare Provision for Asylum-Seekers in Germany

 The following sections briefly outline asylum-seekers’ access to healthcare in Germany. For a detailed account of Germany’s complex health policies for asylum-seekers, please refer to Gottlieb and Schülle.^3^

###  The National Level: Defining the Scope of Health Entitlements

 Asylum-seekers’ social and health benefits are regulated by the federal Asylum Seeker Benefits Act (AsylbLG). Implementation of the AsylbLG – and thus the organization of healthcare provision – is at the discretion of the local governments.^3^

**Figure 1:**
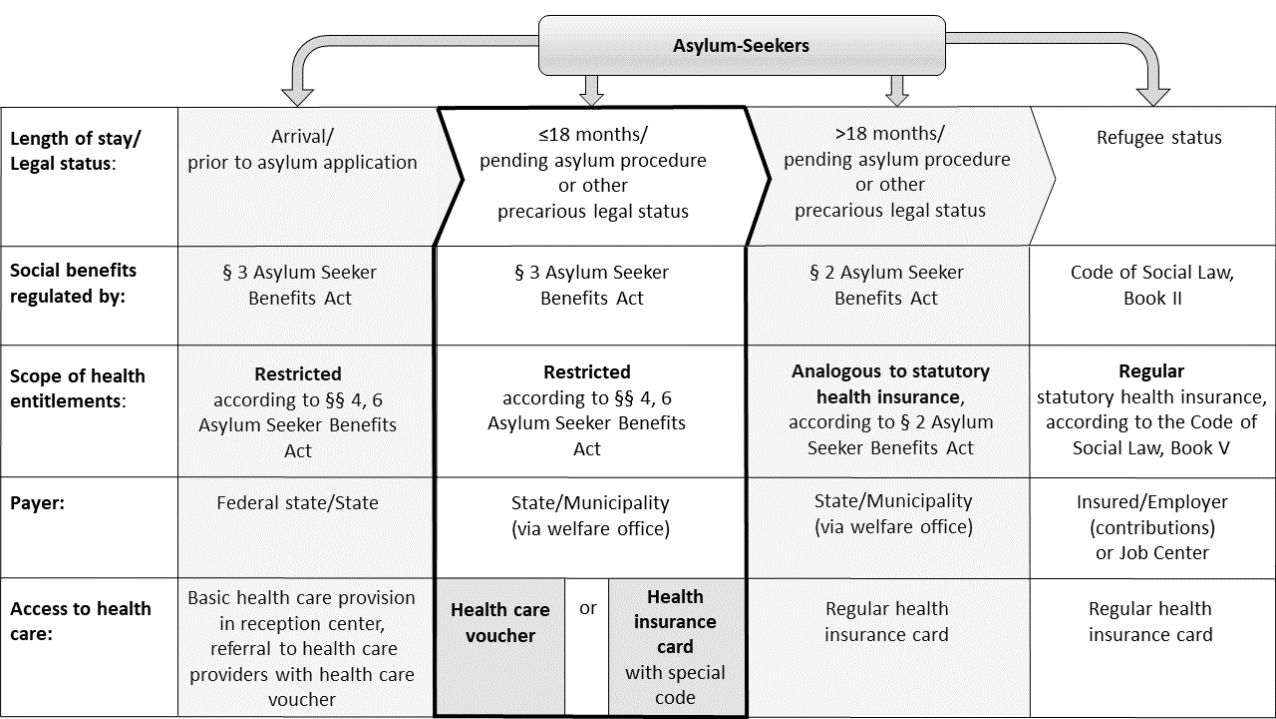
Overview of Asylum-Seeker Health Policies in Germany (source: Gottlieb and Schülle^3^).

 The AsylbLG was introduced in 1993 as a measure of cost containment and migration deterrence. It detached the provision of social and health benefits for asylum-seekers from statutory social and health insurance, thus creating a parallel system; and it introduced a waiting period – currently 18 months – during which a reduced scope of social and health entitlements applies.^3,4^ After 18 months (or upon receiving refugee status), most asylum-seekers become eligible for social and health benefits equivalent to statutorily insured persons. Figure 1 provides an overview of asylum-seeker health policies in Germany, including relevant legal provisions, the scope of health entitlements and access to healthcare, across the different phases of the asylum determination procedure. The subject matter of this study – the introduction of the HIC and its health system effects – is relevant to the 18 months waiting period (central column in Figure 1).

 During the 18 months waiting period, asylum-seekers’ scope of health entitlements is restricted to cases of “acute disease and pain” (AsylbLG §4, 1), the array of preventive services and immunizations that is recommended as part of statutory health insurance, and the full range of maternal and delivery services. Additional services can be granted on a case-by-case basis, if deemed indispensable.^3^

###  The Local Level: Providing Access to Healthcare

 The actual healthcare provided for asylum-seekers varies between states and municipalities.^3,5^ One variation is the employment of different mechanisms to regulate access to healthcare; namely, the use of HVs as opposed to electronic HICs as proof of eligibility. In 2015 only two city states, Bremen and Hamburg, were using HICs.^6^ By 2019, three additional states had comprehensively introduced the HIC. In four states, the HIC had been introduced by some municipalities, while others continued using HVs. Six state governments had ruled the HIC out.^6,7^

 Where HVs are used as proof of eligibility, asylum-seekers must obtain a HV from the welfare office before they can access healthcare (except for emergency care, which can be sought without prior authorization). HVs are valid for three months within the same administrative district. Each referral requires a new HV. Welfare officers decide over medical necessity on a case-by-case basis. Healthcare providers bill the welfare office for the services provided. The welfare offices assume accounting and financial controlling tasks, or they outsource them against an administrative fee to a Sickness Fund. Eventually, the welfare offices reimburse the healthcare providers. This may include the settlement of conflicts; for example, over services that were provided but not reimbursed for upon a negative controlling assessment (for instance, because they exceed the legal scope defined by the AsylbLG), or over recourse claims for erroneous reimbursements.

 Where the HIC is used, contracted Sickness Funds issue the HICs to the asylum-seekers, who can thus access health services in the same way as statutorily insured persons. Healthcare providers, in turn, use the same billing procedure as for statutorily insured patients, vis-à-vis the Sickness Funds. The Sickness Funds carry out the billing and accounting in a similar fashion as for statutorily insured persons (yet within the scope of services as defined by the AsylbLG), against an administrative fee. They transmit the billing information to the welfare offices, which settle the invoices.^3^

###  The Political Debate: Arguing for/Against the HIC

 In political debates on asylum-seekers’ access to healthcare generally, and on the HIC introduction in specific, economic arguments play a central role. Opponents of the HIC claim that it will give rise to an upsurge in medical expenses. Repealing the HV system is seen as giving up an indispensable means of control over access to healthcare, which is assumed to result in an overuse of outpatient health services and to thus raise costs. Moreover, easier access to health benefits is considered an incentive for unsolicited migration, which is framed as an additional burden on the social and health system.^2,8^ Another point of critique is that the HIC incurs higher administrative fees payable to the Sickness Funds than under the HV system (in Berlin, for example, 6% of medical costs, as compared to the previous 5% rate).^9^

 Proponents of the HIC argue that it simplifies bureaucratic procedures and thus reduces administrative costs.^10^ They further note that timely access to outpatient care can hold economic benefits; for example, due to the early detection and treatment of ambulatory care sensitive conditions in the outpatient sector.^11^ This is supported by a growing body of research.^11-14^ The main arguments put forward in favour of the HIC, however, are of normative nature, as they relate to governments’ legal and moral duty to provide equitable and non-discriminatory access to healthcare.^5,15,16^

 Our study uses the recent introduction of the HIC in Berlin as an opportunity to empirically test some of the above described arguments. To this end, we examine effects of the HIC introduction on the local health system. (Implications for asylum-seeking patients are described elsewhere).^17^ We specifically aim to answer the question, whether the HIC leads to changes in terms of cost control and containment.

## Methods

 This mixed-methods study combined quantitative and qualitative methods in a convergent parallel research design to capture a broad range of possible implications related to the HIC introduction, including economic, organizational and ethical effects. The study’s quantitative component consists of a cost analysis and the qualitative component of key informant interviews. Data collection and analysis for the two components was conducted simultaneously, with the goal of expanding knowledge on different, equally important facets of our research question.^18^ Integration of the different component occurred through the merging of quantitative and qualitative findings for comparative interpretation and discussion.^19^

 To our knowledge, this is the first mixed-methods assessment of the local mode of asylum-seeker healthcare provision in Germany, which captures various health system effects in one setting and compares them in a chronological perspective; that is, before and after the HIC introduction. Previous studies applied quantitative cross-sectional study designs^20-22^; that is, some of them rely on comparisons across different settings, which implies certain limitations (see, eg, Wenner et al).^23^ While these studies have generated valuable evidence, quantitative methodologies can be limited in their capacity to address a multifaceted research question; for instance, to produce insights into the whys and hows of complex systems and processes, or into their meaning for different stakeholders.^18,19^ Our study’s examination of one setting through a combination of quantitative and qualitative methods contributes to filling this gap.

###  Cost Analysis

 Thecost analysisused two kinds of information on medical expenses for asylum-seekers: (*a*) claims data from the central financing and controlling department of the Berlin Senate Administration for Integration, Work and Social Affairs (SENIAS), and (*b*) information on expenses for humanitarian healthcare provision by the Berlin State Office for Refugee Affairs (LAF).

 The SENIAS’ claims data included aggregated information on expenses for asylum-seekers’ healthcare during the 18 months waiting period. We retrieved the total annual expenses for administration, inpatient care, and outpatient care for two years before (2014, 2015) and three years after the HIC introduction (2017, 2018, 2019). These budgetary items were chosen because comparable data were available for the period before and after the HIC introduction. In addition to retrieving the claims data itself, we obtained the results of cost controlling procedures, which were implemented together with the HIC in 2016. This information allows for insights into potential patterns (for example, the accumulation of outstandingly high costs in a specific medical area) or individual high-cost cases that have driven cost developments in the respective years.

 To obtain average costs per person per year, we used the number of asylum-seekers in the phase of the 18 months waiting period for the same year as a denominator. Cost data for the year 2016 were removed from the calculation. During this year, both the HV and the HIC system operated in parallel, while the HIC was gradually being rolled out. It is therefore impossible to calculate costs per person and assign them to one of the two access mechanisms (HV or HIC).

 The latter data (*b*) refers to a special LAF budget for the humanitarian provision of primary healthcare through the operation of dedicated walk-in clinics (Medipoints) in the years 2016 to 2018. We added the amounts spent on humanitarian healthcare to the expenses for outpatient care as calculated from the SENIAS claims data for the respective years.

 The cost analysis tests the hypothesis that easier access to healthcare induces high service utilization and thereby raises medical costs. If this hypothesis is true, then we anticipate the HIC introduction to be followed by increasing medical expenses primarily for outpatient care (but not for inpatient care, because access to inpatient services is regulated by health system-inherent gatekeepers). If the hypothesis is false, we predict no significant changes in medical costs; or – as an alternative hypothesis – increased expenses for outpatient services and, at the same time, decreased expenses for inpatient services (due to the early detection and treatment of ambulatory care sensitive conditions in the outpatient sector and a related reduction of avoidable emergency room visits and hospitalizations).

###  Qualitative Interview Analysis

 Semi-structured in-depth interviews with 12 key informants explored effects of the HIC introduction on organizational processes and on ethical dilemmas for staff. Specifically, interviewees were asked to explain work processes related to asylum-seeker healthcare provision before and after the HIC introduction, to recount practical and ethical challenges, to describe changes related to the HIC introduction, and to comparatively evaluate the HIC and the HV. The interview guide had been developed from the existing academic and grey literature on asylum-seeker healthcare provision in Germany. During the interviews, the interview guide was handled flexibly to facilitate a natural flow of conversation and to allow the participants to bring up additional topics.

 Participants were purposely chosen to capture the perspectives of particularly knowledgeable policy and practice stakeholders. They included two representatives of the SENIAS (in one joint interview), two representatives the SENIAS financing and controlling department (in one joint interview), one representative of the LAF, two welfare officers, two hospital administrators, two healthcare providers, and one social worker. In the sectors of health authorities and administration, we were able to interview the key persons involved in asylum-seeker healthcare provision in Berlin and thus achieved data saturation with a relatively low number of interviews. As regards the perspectives of welfare officers and healthcare providers, additional interviews would have been desirable. Yet, formal and time constraints from the part of potential participants hampered the acquisition of additional interviewees (10 additional persons were invited but declined participation).

 Interviews were conducted by one, in most cases two of the authors in the participants’ workplaces. They were between 45 and 80 minutes in length and in German language. Interviews were audio-recorded, transcribed and analyzed based on Mayring’s approach to structuring content analysis.^24^ We used a set of pre-existing codes and categories, which was based on the literature and on informal conversations with practice stakeholders that took place in preparation for this study. They related to work processes, interactions between different stakeholders under the HV and the HIC system respectively, HIC-related changes in administrative workload, practical challenges with the HV and the HIC, ethical dilemmas, evaluations of the HV and the HIC, and suggestions for improvements. At the same time, the analysis remained open to further themes as they emerged from the data. For instance, aspects of financial transparency and security, and claims for more profound policy change were brought up by participants. ATLAS.ti software^25^ was used to code the texts. After one of the authors had completed the analysis of the first four interviews, the coding scheme was discussed among the research team, jointly revised, and then applied to the texts in a final round of analysis. Initial results were discussed with SENIAS and LAF representatives and their feedback integrated in the results presented here (respondent validation). Quotes have been translated from German into English by the authors.

## Results

 The effects of the HIC introduction on the local health system can be categorized into four overarching themes: (*a*) costs, (*b*) financial control and security; (*c*) organizational and work processes; and (*d*) ethics. The following sections will present our findings concerning each theme, with the first section drawing primarily on the results of the cost analysis; whereas the latter three sections rely exclusively on findings from our qualitative interview analysis.

###  Costs

 Many study participants stated that *“[t]he costs per asylum-seeker did not rise [since the HIC introduction] - and this is without factoring in the [reduced] administrative costs”* (LAF representative). On the contrary, they described the previous barriers implied in the HV system as *“shortsighted” *(healthcare provider), explaining that inadequate access to primary healthcare would ultimately increase health needs and expenses: “[*A]t some point, those people, whose [healthcare] access is made so difficult, end up here [in hospital] as an emergency*” (healthcare provider).

**Figure 2:**
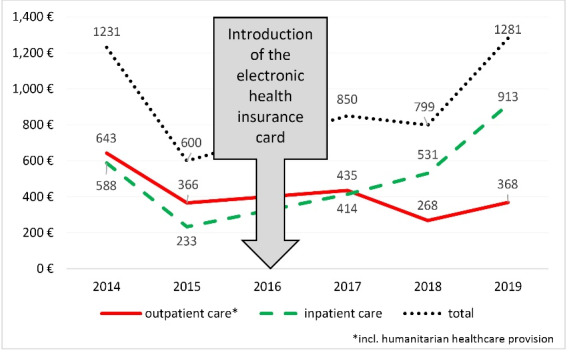
Average Medical Expenses (Per Person/Year) for Asylum-Seekers, Before and After the Introduction of the Electronic Health Insurance Card in Berlin (in €).

 Our cost analysis only partly supports this assessment. In 2014/2015, before the HIC introduction, the total expenses for the provision of inpatient and outpatient care for asylum-seekers average 915€ (SD ±315) per person per year. After the HIC introduction, they rise to an average yearly amount of 977€ (SD ±217). The expenses for outpatient and inpatient care, however, each show divergent trends (see Figure 2).

**Figure 3:**
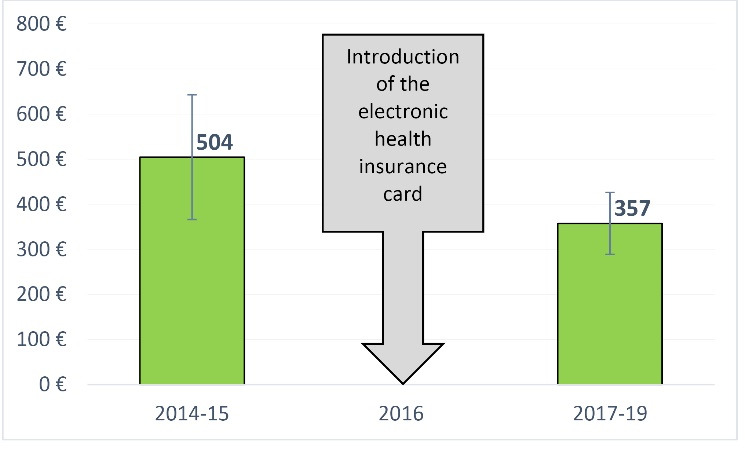
Average Expenses for Outpatient Care (Per Person/Year) for Asylum-Seekers, Before and After the HIC Introduction in Berlin (in €). Abbreviation: HIC, health insurance card.

 The average expenses for outpatient care decrease after the HIC introduction (see Figure 3). When HVs were used, the yearly expenses for outpatient care were 504€ (SD ±138) per person; whereas, since the HIC introduction, they are almost 30% lower at 357€ (SD ±69) (see Figure 3).

**Figure 4:**
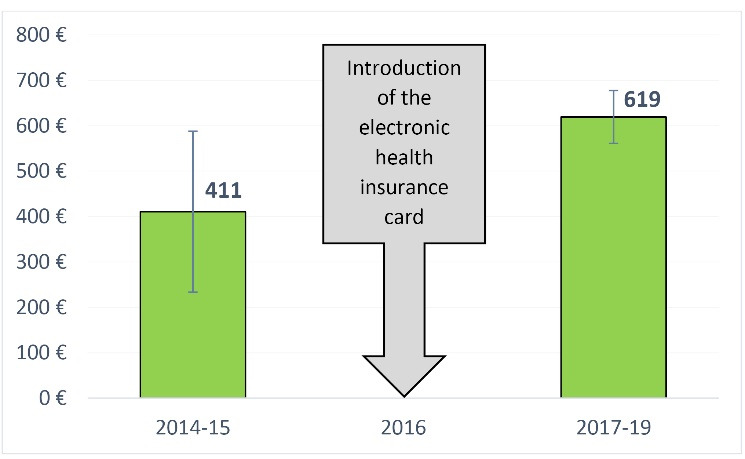
Average Expenses for Inpatient Care (Per Person/Year) for Asylum-Seekers, Before and After the HIC Introduction in Berlin (in €). Abbreviation: HIC, health insurance card.

 The yearly per capita expenses for inpatient care, on the contrary, increase from 411€ (SD ±177) to 619€ (SD ±58) (see Figure 4). According to the respective cost controlling procedures, this increase is related to a rise in the numbers of cases with “medium” hospitalization costs of 20 000-100 000€. The number of individual high-cost cases (that is, cases that incur hospitalization costs of over 100 000€) has remained constant.

 The administrative costs per person rise from a yerly average amount of 41€ (SD ±11) before the HIC introduction to 63€ (SD ±14) after the HIC introduction.

 The year 2015 stands out with low per capita expenses across all cost categories. In comparison to the previous year, average expenses for inpatient care drop from 588€ to 233€, expenses for outpatient care drop from 643€ to 366€, and expenses for administration drop from 51€ to 30€.

###  Financial Control and Security

 Cost control and financial security emerged as important topics from our qualitative interview analysis. Participants noted that the HIC introduction had enabled unprecedented financial transparency, which allowed for improved cost control. Moreover, the HIC had reduced financial insecurities and losses for both the health administration and healthcare providers.

 Representatives of the health administration emphasized that they had had few means of identifying and tracing back accounting errors under the HV system. With a Sickness Fund subcontracted for accounting for outpatient care, they had received summative reimbursement claims per calendric period, rather than detailed cost statements. By way of comparison, “*now [, with the HIC,] we have case-related claims data!” *(SENIAS representative) This also enabled the establishment of software-aided *“controlling procedures. If the [amounts] … billed for are very high, we can now check if everything is correct” *(SENIAS representative).

 Interviewees further noted that it was now possible not only to detect incorrect billings, but also to effectively claim for recourse. They described that, before the HIC introduction, healthcare providers frequently ignored demands to pay back erroneous payments: *“Of course! They already got their money!”* (SENIAS representative) The Sickness Funds that now assume responsibility for accounting, however, have a standard recourse mechanism, which offsets excess payments with the following bills from the same healthcare provider.

 Healthcare providers, too, reported that the HIC has mitigated financial insecurities and losses, which had been implied in the previous HV system. They recounted instances under the HV system, in which they had had great difficulties to get reimbursed for their services, or in which they did not get reimbursed at all. *“[W]e could not bill for at least 10% [of the services that] … we had already provided” *(healthcare provider).They noted that *“sometimes [this happened because] they [asylum-seeking patients] had no [healthcare] voucher. Neither did they know how to get a voucher” *(healthcare provider). In many other instances, they explained, financial losses were related to the HV system’s authorization and controlling procedures, which remained unpredictable for them as healthcare providers. As a result, reimbursement claims were sometimes post-hoc rejected by the welfare office, and healthcare providers had to file objections in a tedious bureaucratic procedure. The HIC introduction has reduced such financial insecurities and losses, insofar as healthcare providers now claim reimbursement vis-à-vis the Sickness Funds in a standard procedure that is familiar and predictable.

###  Organizational and Work Processes

 Most interviewees saw it as an advantage of the HIC that it allows using the same standardized and digitalized work procedures as for statutorily insured patients. Healthcare providers noted that receptionists now only needed to swipe the HIC, instead of manually filling out forms. They appreciated that the billing procedure did not require extra bureaucratic effort from them, unlike the previous HV: “*Now there is simply no difference in the amount of time spent [as compared to statutorily insured patients]*” (healthcare provider). Welfare officers and social workers similarly described that the HIC had simplified their work. Welfare officers emphasized that it had become unnecessary for asylum-seekers to visit the welfare office without appointment to obtain a HV in case of an acute health need. This has made their work more predictable and freed capacities to address other needs of their clientele.

 It was further noted by various participants that *“[ i ]t was unbelievably laborious to employ [the HVs] without any errors. […] The HIC changed this dramatically” *(social worker). Previously, the welfare officers had been required to fill out different HVs for different healthcare providers with all the details required for the particular occasion; whereas now the relevant information *“is always stored there [on the HIC]. … And then I know that things work smoothly”* (social worker).

 Even when filled out correctly, *“the HVs were valid only for a limited time [three months]. And this made the arrangement of appointments additionally difficult” *(social worker); because it could happen that, by the time of the appointment, the HV had lost its validity. As a result,appointments had to be cancelled and new ones arranged; or *“[one] had to tell [the asylum-seeker] ‘Okay, now you need yet another [HV]’” *(social worker). The HVs’ validity restrictions had thus requiredpatients, social workers and/or healthcare providers to invest extra efforts and time in order to actualize a doctor’s appointment.

 Some interviewees did not see significant changes in their workload. LAF and welfare officers noted that frequent changes in asylum-seekers’ personal details (for example, address and name spelling) each time required the issuing of a new HIC. Furthermore, it was pointed out that *“[t]he effort [for introducing the HIC] was extremely high” *(SENIAS representative), as it implied the establishment and familiarization with a new electronic system. However, interviewees also noted that the digitalization of the Senate Administration’s work processes had been overdue, regardless of the issue of asylum-seekers’ health service provision: *“It is a lot of work to change everything and establish something new. But that’s what it takes to be up to date … Actually, the Senate should be thankful [for the opportunity], because anyhow it was time to supersede the old [HV] system”* (SENIAS representative). From this perspective, the arrival of large numbers of asylum-seekers and the subsequent administrative crisis have functioned as a catalyst for long needed reform of organizational procedures.

###  Ethics

 Our interviewees discussed a broad range of ethical issues in asylum-seeker healthcare provision. Most of these issues primarily affect patients (for instance, questions related to health equity and stigmatization). At the same time they can generate burdens on the health system, when frictions with agreed-on principles create dilemmas for staff.

 For instance, healthcare providers recounted that, under the HV system, *“you sometimes spent months trying to get 40€. And … the doctors said: ‘I won‘ t do that. […]’ Economically it is … absolute nonsense”* (healthcare provider). Hence, the bureaucratic efforts and financial insecurities implied in the HV system deterred physicians from accepting asylum-seeking patients. They were thus liable to put medical professionals in a double bind between legitimate financial interests and professional-ethical codes, which command impartial and non-discriminatory provision of medical care.

 A lot of criticism related to the ways in which responsibilities for decision-making on a person’s access to healthcare had been allotted under the HV system. Participants particularly disapproved of the fact that administrative staff had to judge over medical necessity (as part of the HV issuing procedure) and over adequacy of treatment (as part of cost controlling). They noted that the HIC, in contrast, places the decision over medical necessity in the hands of medically qualified healthcare providers; and it makes *“the Pros, that is, the Sickness Funds, take care [of the accounting]”* (LAF representative). This was regarded as a better matching of responsibilities and competencies. At the same time, it releases welfare officers of the duty to handle asylum-seekers medical information, which had brought them into conflict with patient confidentiality and data protection standards.

 Finally, interviewees described the HV system as an overt contradiction to the Berlin Senate’s commitment to equity, non-discrimination and integration, in that it erected *“insanely high barrier[ s to care]” *and implied stigma and inequality: *“[The HV] was green and with a big, black A on it, for asylum. To make obvious which clientele this is about” *(LAF representative). By way of comparison, the HIC was appreciated as *“a step toward integration”* (healthcare provider). Hence, to a certain extent, the HIC has helped reconcile de facto healthcare provision for asylum-seekers with the ethical and legal principles that most healthcare administrators and providers feel committed to.

 However, it was also pointed out that the most fundamental contradiction with health equity principles has remained untouched by the HIC introduction; namely, the federal legal framework for asylum-seekers healthcare, which stipulates the regulation and administration of restricted health benefits through a parallel system, alongside statutory social and health insurance. Interviewees suggested that *“one should actually change the [federal] Asylum Seeker Benefits Act” *(SENIAS representative). From this perspective, the HIC introduction was a positive measure to “patch up*”* the existing system of asylum-seeker healthcare provision. Yet, as such it also signifies a missed opportunity for systemic change, which could have contributed to greater health equity for asylum-seekers in a more comprehensive fashion.

## Discussion

 Our study aimed at contributing empirical evidence to the discussions on asylum-seeker health policies in Germany. To this end we examined health system effects of the HIC introduction in Berlin. Acknowledging the central role of economic arguments in the political debates on asylum-seekers’ access to healthcare, we focused on aspects of cost control and containment.

###  Does the HIC Compromise Cost Control and Containment?

 Our results show that the per capita expenses for outpatient services have declined in the wake of the HIC introduction. This is despite the inclusion of the costs of humanitarian healthcare provision in the calculation, which has rendered our estimates conservative. Other authors similarly report that the HIC introduction has not led to an overuse of ambulatory health services in other German states.^22^ They rather find evidence for broader, more needs-based utilization of outpatient healthcare across the asylum-seeking population, alongside decreasing costs for outpatient care provision.^20^ Hence, the hypothesis that the removal of access barriers for asylum-seekers will induce unduly high service utilization and thus raise medical costs can be rejected.

 Based on the existing literature, we had anticipated that improved access to outpatient care may help reduce inpatient care utilization (alternative hypothesis). Different studies consistently report high utilization rates of inpatient services among asylum-seekers, including avoidable hospitalizations.^12,20,21,23,26^ Some authors have suggested that improved accessibility of outpatient services could help bring these rates down through early detection and treatment of ambulatory care sensitive conditions in the outpatient sector, and thus decrease costs in the inpatient sector. However, this alternative hypothesis is neither supported by our findings: In our study, expenses for inpatient services appear to be on the rise. This rise in costs is due to a rise in case numbers and not a few high cost-cases.

 One possible explanation is a backlog of cases, related to low accessibility of healthcare for asylum-seekers during the administrative crisis in 2015. Together with lengthy administrative and billing procedures, this may have carried costs for inpatient care provision over into the following years. Another explanation for rising inpatient care expenses is that more inpatient procedures get authorized under the HIC system. This could mean that the coverage of procedures, after being recommended by the treating physician, was more frequently rejected as long as welfare officers executed authorization and controlling tasks under the HV system, as compared to authorization and controlling via the Sickness Funds under the HIC system. In that case, high utilization and costs for inpatient care may reflect more needs-based healthcare provision, in line with vertical health equity principles (different healthcare access in accordance with different health needs).^20,27^ Yet another explanation for consistently high utilization and costs of inpatient services among asylum-seeking populations are remaining barriers to healthcare, such as language barriers, unfamiliarity with the health system, and the lack of culturally sensitive approaches to diagnostics and treatment. Such barriers may continue to hamper the timely provision of effective treatment in the outpatient sector, thus leading to delays, complications and ultimately to avoidable emergency room visits and hospitalizations. To understand the reasons for persistently high hospitalization rates among asylum-seeking populations in Berlin, further research is required.^28,29^

 The HIC introduction implied labor- and investment-intensive system changes. The rise in administrative costs that showed in our study may, to some extent, reflect such investments. Our results thus do substantiate the concern that introducing the HIC may not be cost-effective for small municipalities and administrative districts.^2^ The frequent relocation of asylum-seekers during the asylum determination procedure creates additional administrative workloads, as new HICs must be issued with each relocation. Hence, both from an administrative and a health perspective, more stable living conditions for asylum-seekers would be desirable.

 The year 2015 stands out in our cost analysis with low expenses across all cost categories. Importantly, that year was characterized by the arrival of large numbers of asylum-seekers and related shortfalls in social and health service provision. Low expenditures in that year, therefore, must be understood as a reflection of insufficient access to healthcare, rather than a desirable achievement and point of reference.

 Our qualitative results point to unprecedented financial transparency and cost control as major advantages of the HIC. This is remarkable, not least because the main argument for introducing the AsylbLG and the HV system in 1993 was the desire to control and contain expenditures for asylum-seekers’ social and health needs.^30,31^ Paradoxically, from our study, the HV system emerges as flawed in terms of financial transparency and cost control. In contrast, the HIC system provides a detailed breakdown of healthcare-related costs per case; and it thus enables effective controlling measures. Effective cost controlling, in turn, helps prevent accounting mistakes amounting to 5%-10% of overall health expenditures, according to the Sickness Funds.^7^ Such cost savings should be taken into account when considering the economic effects of the HIC introduction in policy debates.

 Overall, a growing body of evidence, including this study, challenges the frequent argument in migrant health policy debates that formal restrictions were a necessary means to prevent overuse of health services and safeguard public resources. Perpetuating this argument despite better knowledge not only harms migrant communities; it also distorts the debate and deceives the public.^32^ More correctly, policy-makers should communicate to their electorate that erecting barriers to healthcare entails extra costs and burdens on the health system, rather than saving resources. This way, the public should be enabled to make an informed choice whether it is willing to pay this price for the purpose of restricting asylum-seekers’ healthcare access.

###  What Other Changes Did the HIC Effect for Berlin’s Health System?

 Our study points to manifold improvements for medical and administrative staff as a result of the HIC introduction. These include simplified and predictable work processes and less ethical dilemmas. In line with previous research,^33-35^ our study underscores that restrictions on marginalized groups’ healthcare access confront health professionals with practical and ethical predicaments, as they manage paradoxes between legal provisions, professional-ethical codes, financial necessities, and personal values. Other authors have measured HIC-related reductions of administrative workloads in terms of potential cutbacks on personnel costs. The Hamburg health administration, for example, quantified the respective cost reductions with 1.4 million € per year.^10^ Beyond such “hard” monetary value, we suggest considering “soft” organizational gains. For instance, the mitigation of dilemmas may contribute to the reduction of occupational stress among health professionals.

 Ultimately, it should be noted that the change from HV to HIC - despite the surrounding political controversies – is a small “tweaking” of the system, which leaves the core of asylum-seeker health policies in Germany untouched. This core is a parallel system with restricted social and health benefits. Our study underscores that local level stakeholders question this policy. In their view, it would make more sense – from administrative, economic and ethical perspectives – to (re-)integrate asylum-seekers in the statutory social and health insurance scheme. Yet, such systemic reform requires changing federal law; and until this happens, amendments of the current system (like the HIC introduction) help local level stakeholders manage its inherent tensions.

###  Limitations

 Our study underlies several limitations. The aggregated claims data that was obtained from the SENIAS allowed for very crude analyses only. Due to differences in the accounting procedures before and after the HIC introduction, we could only compare expenses for inpatient and outpatient services and administration. More detailed stratifications, for example, in different cost categories, are not available for the period during which the HV system was used. A calculation of per capita expenditures was only possible by drawing on the number of recipients of AsylbLG benefits for the same year. Most importantly, the timeframe under consideration was too short to make definite statements about cost developments. However, data from previous years, earlier than 2014, were not available. To facilitate future research on the health needs and healthcare seeking of asylum-seeking populations, the availability, accessibility and quality of data must be improved.

 Our interview participants represent the range of relevant health system stakeholders. Especially in the health authority and health administration sector, we were able to include the perspectives of the most relevant key informants. As regards healthcare providers and welfare officers, a higher number of participants would have been desirable. Yet, time and formal constraints from part of the potential participants hampered the recruitment of additional interviewees. Social and health service providers hold valuable knowledge on how current asylum-seeker health policies play out in terms of the development of local practices of healthcare provision and their context-specific outcomes. Future research should tap into this knowledge to yield a nuanced picture of the reality of asylum-seeker healthcare provision.

 The perspectives included here may be biased toward equitable healthcare provision for asylum-seekers. This could be related to self-selection and social desirability bias or, more likely, to the official stance of the Berlin Senate on social diversity, which often stands out as progressive and inclusive as compared to other German states and to the federal government. Including more controversial perspectives in future studies could be instructive to better understand the variety of motives and perceptions in the political debates on asylum-seeker healthcare.

## Conclusion

 It is a recurrent argument in political debates that asylum-seekers, unless extrinsically controlled, will overuse health services and thereby raise expenses.^32,36^ Our study finds no evidential basis for these claims. On the contrary, the example of the HIC introduction in Berlin indicates that the removal of barriers to healthcare offers economic *and* other benefits to the local health system. The HIC has reduced bureaucratic complexity and administrative workloads; it has enabled unprecedented financial transparency and control; and it has mitigated ethical tensions. These advantages came at no increase in costs for outpatient healthcare provision. On the contrary, the expenses for outpatient healthcare provision for asylum-seekers decline since the HIC introduction. In the inpatient sector, on the other hand, expenses appear to be rising. While this development may indicate needs-based utilization of health services, it warrants further investigation. Overall, our study contributes to a growing body of evidence suggesting that the removal of barriers to healthcare is a more sensible health policy choice than the maintenance of costly control mechanisms.

## Acknowledgements

 The authors wish to thank the SENIAS and its central financing and controlling department as well as the LAF for their support of this study. They extend further thanks to all study participants for their valuable time.

## Ethical issues

 Ethical approval was obtained from the Charité Ethics Committee (EA4/111/18).

## Competing interests

 Authors declare that they have no competing interests.

## Authors’ contributions

 NG has conceived of and planned the study. NG, VO, and MK collected the data, with NG facilitating access to the field. NG, VO, and MK analysed the data. NG led the writing up and subsequent revision of the study results, with VO and MK providing feedback. All authors have approved of the final version of the article.

## Disclaimer

 The views expressed in the submitted article are our own and not an official position of the institution or funder.

## Funding

 The research leading to these results has received funding from the People Programme (Marie Curie Actions) of the European Union’s Seventh Framework Programme (FP7/2007-2013) under REA grant agreement no. 600209 (TU Berlin/IPODI).
